# Reduced protein carbonylation on hormone therapy is associated with improved fibrinolysis in postmenopausal women: the impact of PAI-1 and TAFI activity

**DOI:** 10.1007/s11239-024-03006-w

**Published:** 2024-07-09

**Authors:** Magdalena Piróg, Michał Ząbczyk, Joanna Natorska, Robert Jach, Anetta Undas

**Affiliations:** 1 Gynecological Endocrinology Department, Jagiellonian University Medical College, Krakow, Poland; 2 Department of Thromboembolic Disorders, Institute of Cardiology, Jagiellonian University Medical College, Pradnicka 80, 31-202 Krakow, Poland; 3 Krakow Centre for Medical Research and Technologies, St. John Paul II Hospital, Krakow, Poland

**Keywords:** Fibrinolysis, Hormone replacement therapy, Protein carbonylation, Menopause

## Abstract

Hormone therapy (HT) has been reported to reduce protein carbonylation (PC) in postmenopausal women, in whom fibrinolysis is impaired. We investigated whether PC affects fibrinolysis and if HT modulates this effect. We enrolled 150 women aged 55.5 ± 4.7 years in a randomized interventional open-label study, including 50 on standard oral HT, 50 on ultra-low-dose HT, and 50 controls. PC, along with global fibrinolysis (clot lysis time, CLT), fibrinolysis proteins, and prothrombotic markers were determined at baseline and at 24 weeks. Patients with the baseline top quartile PC (> 2.07 nM/mg protein) had 10.3% longer CLT, higher activity (but not antigen) of TAFI (+ 19.9%) and PAI-1 (+ 68.1%) compared to the remainder. No differences were observed in thrombin generation, factor VIII, plasminogen or α_2_-antiplasmin. On-treatment PC decreased by 16.4% (*p* < 0.0001), without differences related to the type of HT, compared to baseline and by 30% compared to controls, in whom PC and fibrinolysis markers remained unchanged. Patients with PC > 2.07 nM/mg had shortened CLT during HT compared to baseline, along with lower PAI-1 (-69%) and TAFI (-26%) activity. In this subgroup CLT was 5.8% shorter compared to controls with the highest PC. In postmenopausal women with increased PC, HT was accompanied by PC reduction and faster clot lysis together with decreased PAI-1 and TAFI activity.

## Introduction

Menopausal transition is a natural stage in the aging process related to an increased risk of thromboembolic events, which are associated with increased platelet activation, unfavorably altered endothelial function along with modification in blood coagulation profile towards increased thrombotic tendency [1, 2]. Age-related changes in blood coagulation and fibrinolysis are mediated via particular mechanisms, such as enhanced oxidative stress, and changes in the oxidant/antioxidant mechanisms [3]. Increased generation of reactive oxygen species (ROS) leads to enhanced protein carbonylation (PC), the most common and irreversible non-enzymatic post-translational protein modification [4]. Enhanced PC may directly either activate or inactivate specific enzymes as well as markedly alter the protein structure [5]. Fibrinogen has been shown to be highly sensitive to oxidative modifications including carbonylation [6, 7]. Fibrinogen carbonylation has been reported to modify fibrin clot structure, which results in the formation of denser fibrin clots composed of thinner fibers, and impair susceptibility to lysis, which represent the so-called prothrombotic fibrin clot phenotype [7, 8]. Increased PC has been reported to be an important marker of protein oxidation in multiple diseases including diabetes [9, 10], coronary artery disease [11], and multiple sclerosis [12].

Hormone therapy (HT), including estrogens, reduces the climacteric symptoms [13, 14], but it increases the risk of stroke [15], myocardial infarction (MI) [16], and venous thromboembolism [17]. Estrogens by containing a phenol ring may effectively scavenge hydroxyl radicals and, consequently, form hydroxylated products [18]. Growing evidence has shown that estrogens reduce the production of ROS, therefore, cessation in ovarian estrogen production among postmenopausal women is thought to contribute to enhanced oxidative stress [19]. On the other hand, estrogens can indirectly increase the activity of antioxidant enzymes [20].

To our knowledge, there have been 2 published studies on PC in relation to HT in postmenopausal women. One study showed decreased PC levels after both transdermal (-17.5%, *n* = 28) and oral route of HT (-19.7%, *n* = 30) used for 24 weeks when compared with the controls [20]. Another small study (*n* = 12) demonstrated lower PC concentration (-21.1%) after 24-week oral HT [21]. To our knowledge, there have been no published reports to explore whether HT-mediated changes in circulating PC affect a prothrombotic state, including fibrinolysis, after menopause and if HT may alter its markers in part via modulation of oxidative protein modifications. Previously, we have demonstrated enhanced fibrinolysis together with decreased both PAI-1 activity and antigen in the ultra-low-dose HT group when compared with the standard HT group [22]. There is evidence that elevated PC is associated with prolonged clot lysis largely via PAI-1 increase as shown in stroke patients [23]. To evaluate whether associations similar to those observed in stroke can be noted in postmenopausal women with or without HT, we conducted a post hoc analysis of the previous study [22] to investigate whether HT administered in the two regimens, i.e., standard and ultra-low doses, can affect circulating PC levels and fibrinolysis in postmenopausal women.

## Materials and methods

### Patients

We recruited 169 postmenopausal, white women at the tertiary gynecology center. Menopause was defined as amenorrhea for more than 12 months with an intact uterus. The study population was described previously in detail [22]. Briefly, the exclusion criteria were: previous thrombotic events, known malignancy, diabetes mellitus; hypo- or hyperthyroidism, adrenal insufficiency, and current smoking.

We collected basic demographic and clinical data, including concomitant diseases and medication used. Both physical examination and gynecologic assessment were performed at enrollment and after 6 months of therapy. Body mass index (BMI) was calculated by dividing weight in kilograms by square of height in meters. Arterial hypertension, obesity and hypercholesterolemia were defined as previously described [22]. Diabetes mellitus was defined in accordance to the American Diabetes Association Criteria [23]. Smoking was defined as the daily use of 1 or more cigarettes. Heavy menstrual bleeding was defined as previously described [24].

The Ethics Committee of Jagiellonian University Medical College approved the study (Approval no. KBET/347/B/2012) and participants provided informed consent in accordance with the Declaration of Helsinki.

### Study design

In the interventional study we enrolled 150 women who were randomly assigned in an open-label manner (1:1:1) to one of three groups: 1) standard oral HT, containing 17β-estradiol (1 mg/day) and dydrogesterone (5 mg/day; Femoston conti, Abbott, Weesp, Netherlands), *n* = 50; 2) ultra-low-dose HT, receiving 17β-estradiol (0.5 mg/day) and dydrogesterone (2.5 mg/day; Femoston mini, Abbott, Olst, Netherlands), *n* = 50; and 3) control group, who did not use HT, *n* = 50. Compliance with therapy was assessed by the pill count. The intervention lasted 6 months.

### Laboratory Investigations

Venous blood samples were drawn with minimal stasis using atraumatic venipuncture at 08.00–10.00 AM, after an overnight fast. All laboratory investigations were performed at study entry and after 6 months of therapy. Routine laboratory tests including blood cell count, lipid profile, fasting plasma glucose, creatinine, antithrombin activity, thyroid-stimulating hormone, follicle-stimulating hormone (FSH), prolactin (PRL), estradiol, and alanine aminotransferase (ALT) were measured by routine laboratory techniques. Plasma fibrinogen levels were measured using the von Clauss method. High-sensitivity C-reactive protein (CRP) was measured by nephelometry (Siemens, Marburg, Germany). Factor (F)VIII was measured by one-stage clotting assay using factor-deficient plasma (Siemens).

#### Carbonylation assessment

Oxidative modification on plasma proteins was assessed according to the approach by Becatti et al. as previously described [25]. Briefly, plasma (100 μl) was incubated with DNPH (400 μl), precipitated with trichloroacetic acid and washed with a 1:1 ratio of ethanol/ethyl acetate solution. The pellet was suspended in guanidine hydrochloride. The product of the reaction was analyzed at 370 nm. PC was expressed in nmol/mL of PC per 1 mg of protein. In healthy subjects, the reference range, established in our laboratory, was 0.54–2.03 nmol/mg protein.

#### Fibrinolysis proteins

We measured plasma PAI-1 antigen levels (Berichrom PAI, Siemens, Marburg, Germany) and activity (Abcam, Cambridge, UK). Plasma α_2_-antiplasmin and plasminogen activity were measured by chromogenic assays (STA Stachrom antiplasmin and STA Stachrom plasminogen, Diagnostica Stago, Asniéres, France). Plasma TAFI activity was measured by a chromogenic assay using the ACTICHROME® Plasma TAFI Activity Kit (American Diagnostica, Stamford, CT, USA). We also measured activated and inactivated TAFI antigen in plasma (TAFIa/TAFIai; Diagnostica Stago, Asnieres, France) and results were expressed as percentage of pooled plasma from healthy volunteers.

To determine fibrinolysis and thrombin generation, blood samples (vol/vol, 9:1 of 3.2% trisodium citrate) were centrifuged (2000 × g), within 30 min since the blood draw, for 10 min. The supernatant was aliquoted and stored (at –80 °C) until analysis [26]. Analysis was performed by experienced technicians blinded to the sample origin.

#### Thrombin generation

Calibrated automated thrombogram (CAT, Thrombinoscope, BV, Maastricht, Netherlands) was performed using commercial reagents as described previously [22]. Briefly, 80 μL of platelet-poor plasma were mixed with 20 μL of the PPP-reagent and 20 μL of FluCa. We measured the lag phase, the maximum concentration of thrombin formed (the thrombin peak), and the area under the curve represented by the endogenous thrombin potential (ETP).

Prothrombin fragments 1 + 2 (F1 + 2) levels were assessed in citrated plasma using an ELISA (Enzygnost F1 + 2 Micro; Siemens).

#### Fibrin clot lysis

A modified approach introduced by Lisman et al. [27] was used to determine clot lysis time (CLT). Briefly, citrated plasma was mixed with CaCl_2_ (final concentration, 15 mmol/l), recombinant human tissue factor (final concentration of 0.6 pmol/l; Innovin, Siemens), phospholipid vesicles (final concentration, 12 μmol/l), and recombined tissue plasminogen activator (rtPA; final concentration, 60 ng/ml Boehringer Ingelheim, Ingelheim, Germany). The turbidity was measured at 405 nm. CLT was defined as the time from the midpoint of the clear-to-maximum-turbid transition, which represents clot formation, to the midpoint of the maximum-turbid-to-clear transition.

All measurements were determined in duplicate by technicians blinded to the origin of the samples. Intra-assay coefficients variation was 5% and inter-assay 7%.

### Statistical analyses

Statistical analysis was performed with the STATISTICA 13.0 software (StatSoft, Poland).

Categorical variables were presented as numbers and percentages. Continuous variables were expressed as mean ± standard deviation or median and interquartile range (IQR), as appropriate. The Shapiro–Wilk test was used to assess conformity with a normal distribution whereas non-normally distributed data were analyzed by Kruskal–Wallis followed by Mann–Whitney’s comparison test. Categorical variables were analyzed using either the Chi^2^ test or Fisher’s exact test. Pearson’s correlation coefficient (Pearson’s r) or Spearman’s rank correlation coefficient were calculated to assess the linear correlations between variables with a normal or non-normal distribution, respectively. To assess the differences between pre- and post-treatment values of continuous variables the Wilcoxon signed-ranks test was applied. For nominal variables the McNemar’s test was used. Associations between the variables were expressed as odds ratio with 95% confidence intervals. Two-sided *P*-values < 0.05 were considered statistically significant.

## Results

### At baseline

We enrolled 150 women aged 55.5 ± 4.7 (range from 46 to 64 years) years. As many as 122 women completed 24-week observation, while 28 were excluded due to early termination (*n* = 22), thromboprophylaxis used (*n* = 4) or abnormal uterine bleeding (*n* = 2). The three study groups were similar in terms of demographics, comorbidities and routine laboratory investigations, as shown previously [22].

**Fig. 1:**
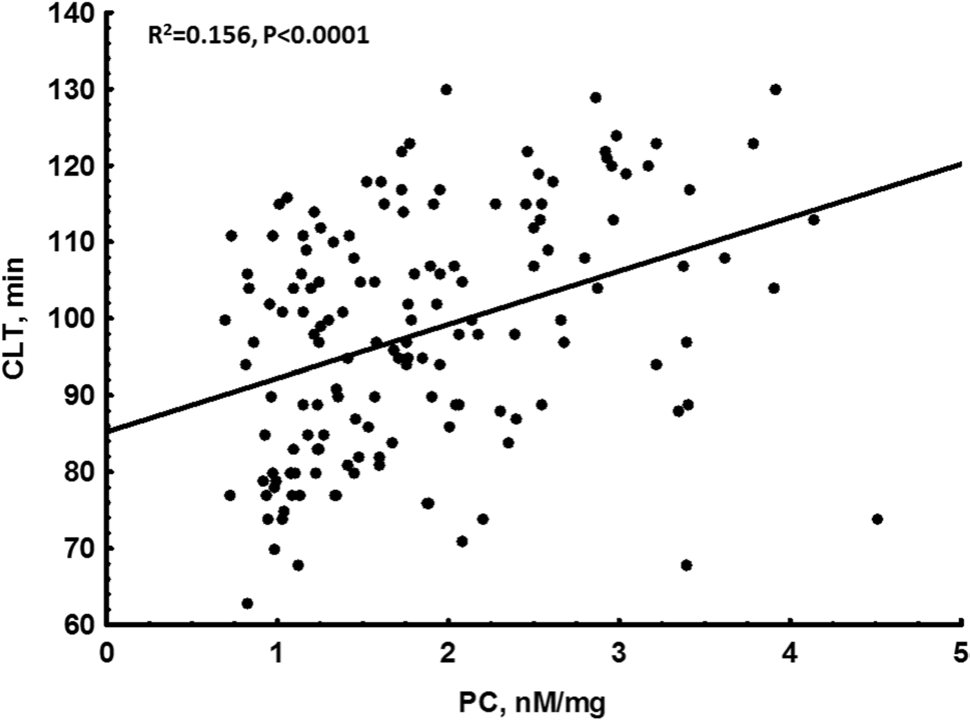
An association between clot lysis time (CLT) and protein carbonylation (PC) at baseline in all postmenopausal women

At baseline, a median PC concentration in the whole cohort was 1.68 nM/mg protein with a range from 0.69 to 5.56 nM/mg protein. Given the upper limit of our reference range (2.03 nM/mg protein), 52 (34.2%) women had elevated PC, including 23 controls. There were no differences in PC levels between patients assigned to the HT groups and controls (1.59 [1.16–2.07] vs. 1.84 [1.27–2.61] nM/mg, *p* = 0.10). PC showed no associations with demographics, routine laboratory variables or hormones concentrations (all *p* > 0.05). In all postmenopausal women PC correlated positively with CLT (Fig. 1). No similar associations were noted between PC and PAI-1 or TAFI (antigen and activity), fibrinogen, FVIII, plasminogen, α_2_-antiplasmin, or thrombin generation using 2 assays (all *p* > 0.05).

As shown in Table 1, patients with the highest PC (> 2.07 nM/mg protein; the top quartile) at baseline compared to the remainder had prolonged CLT (+ 10.3%; significant, also after adjustment for fibrinogen; *p* = 0.009) along with higher activity (but not antigen concentrations) of the two key fibrinolysis inhibitors, i.e., TAFI (+ 19.9%) and PAI-1 (+ 68.4%). There were no similar differences in α_2_-antiplasmin or plasminogen, however the latter variable tended to be lower in women with PC in the top quartile (Table 1). There were no differences in lag phase, peak thrombin generated or ETP as well as in F1 + 2 in relation to baseline PC (Table 1).

**Table 1:** Baseline characteristics of menopausal women depending on protein carbonylation (PC)

Variables	Top quartile (PC > 2.07 nM/mg protein) (*n* = 25)	first-third quartiles (PC ≤ 2.07 nM/mg protein) (*n* = 75)	*P*-value
Age, years	55.3 ± 4.3	56.1 ± 2.2	0.22
BMI, kg/m^2^	25.4 [23.4–27.5]	25.7 [23.5–27.1]	0.10
Routine laboratory investigations			
Hemoglobin, g/dL	13.4 [12.4–13.8]	13.3 [12.7–13.7]	0.86
WBC, × 10^9^/L	6.3 [4.9–7.7]	5.6 [5.1–6.0]	0.93
RBC, × 10^3^/µL	4.6 ± 0.3	4.1 ± 0.9	0.41
Platelets, × 10^9^/L	267 [223–320]	267 [215–291]	0.74
Fasting glucose, mmol/L	5.2 [5.1–5.5]	5.1 [4.8–5.5]	0.13
TSH, µIU/mL	1.6 ± 0.9	1.6 ± 0.7	0.22
FSH, mIU/mL	49.9 [9.3–63.2]	56.8 [37.5–85.4]	0.76
PRL, µIU/mL	206 [161–259]	238 [179–293]	0.73
Estradiol, pM	82 [46–390]	83 [40–181]	0.31
ALT, U/L	18 [14–30]	20 [15–21]	0.46
Creatinine, µM	69 [61–77]	65 [58–77]	0.32
hsCRP, mg/L	1.8 [0.7–3.1]	2.0 [0.8–2.7]	0.43
TC, mM	5.3 [4.6–6.2]	5.6 [4.7–6.1]	0.22
HDL-C, mM	1.5 [1.1–1.9]	1.6 [1.4–2.1]	0.56
LDL-C, mM	3.4 [2.5–4.3]	3.3 [2.4–3.8]	0.50
TG, mM	1.2 [0.7–1.7]	1.0 [0.8–1.8]	0.45
Hemostatic variables and fibrin clot properties			
Antithrombin, %	90 [85–102]	92 [86–99]	0.38
Fibrinogen, g/L	3.6 [3.1–4.1]	3.5 [2.9–3.9]	0.97
FVIII, %	125 [112–156]	130 [116–149]	0.78
Plasminogen activity, %	97 [88–103]	101 [96–127]	0.06
PAI-1 activity, IU/mL	13.3 [7.9–20.2]	7.9 [4.8–11.4]	0.004
PAI-1 antigen, ng/mL	26.4 [20.4–28.9]	27.3 [24.5–29.6]	0.64
TAFI activity, µg/mL	21.3 [18.2–24.2]	17.8 [15.2–20.2]	0.04
TAFI antigen, %	103 [98–122]	110 [89–125]	0.53
α2-antiplasmin, %	101 [89–112]	103 [89–113]	0.84
CLT, min	107 [97–117]	97 [82–107]	0.0084
F1 + 2, pM	253 [223–301]	244 [199–292]	0.22
Lag phase, min	2.7 [2.3–3.2]	3.0 [2.6–3.5]	0.15
Peak thrombin, nM	266 [227–306]	286 [238–330]	0.37
ETP, nM × min	1593 [1346–1700]	1529 [1407–1669]	0.96
PC, nM/mg protein	2.83 [2.46–3.36]	1.40 [1.04–1.82]	< 0.0001

Data are shown as median (IQR) or number (percentage)

*BMI* body mass index, *WBC* white blood cells, *RBC* red blood cells, *TSH* thyroid-stimulating hormone, *FSH* follicle-stimulating hormone, *PRL* prolactin, *ALT* alanine aminotransferase, *hsCRP* high-sensitivity C-reactive protein, *TC* total cholesterol, *HDL-C* high-density lipoprotein cholesterol, *LDL-C* low-density lipoprotein cholesterol, *TG* triglycerides, *FVIII* factor VIII, *PAI-1* plasminogen activator inhibitor-1, *TAFI* thrombin activatable fibrinolysis inhibitor, *CLT* clot lysis time, *ETP* endogenous thrombin potential, *F1 + 2* prothrombin fragment 1 + 2, *PC* protein carbonyl

### At a 24-week follow-up

**Fig. 2:**
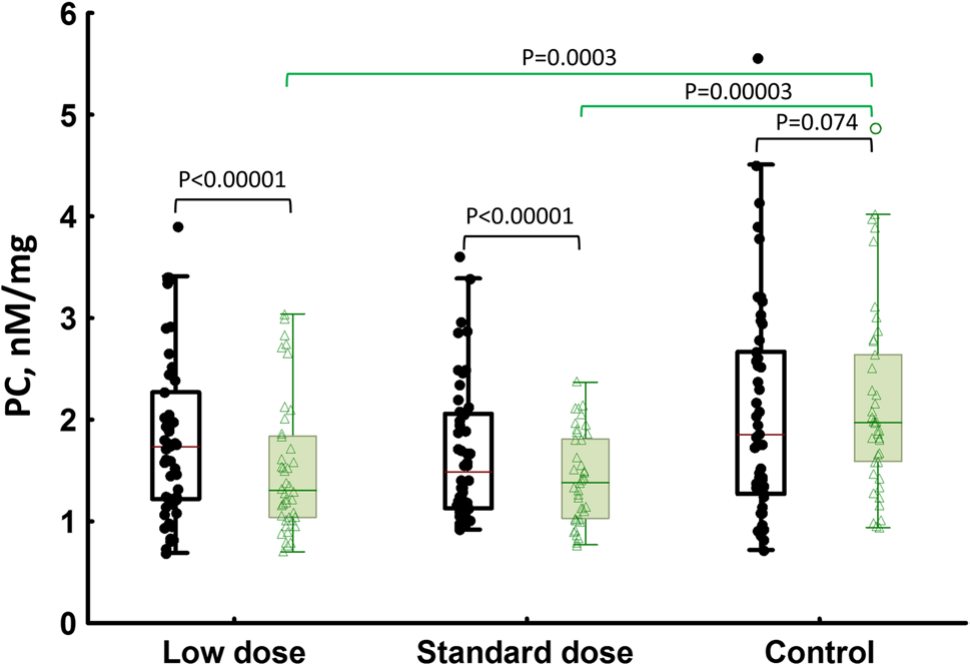
Protein carbonyl (PC) levels at baseline (in black) and at the end of the study (in green) in women on ultra-low-dose hormone therapy (HT), standard HT, and controls

All HT treated women had 32.5% lower on-treatment PC levels compared to the untreated group (1.33 [1.03–1.84] vs 1.97 [1.59–2.64] nM/mg protein, *p* < 0.0001), in which PC levels remained unchanged (Fig. 2). Of note, both ultra-low-dose and standard HT lowered PC levels by 35% and 30%, respectively, as compared to controls (Fig. 2). On-treatment PC levels compared to baseline values were decreased by 16.4% (1.59 [1.16–2.07] vs. 1.33 [1.03–1.84] nM/mg protein, *p* < 0.0001), without significant differences between the two HT groups (Fig. 2). After 24 weeks of HT use, 13 (15.7%) women, including 5 (12.2%) in the standard HT group had PC above the upper limit of our reference range for healthy subjects.

**Fig. 3:**
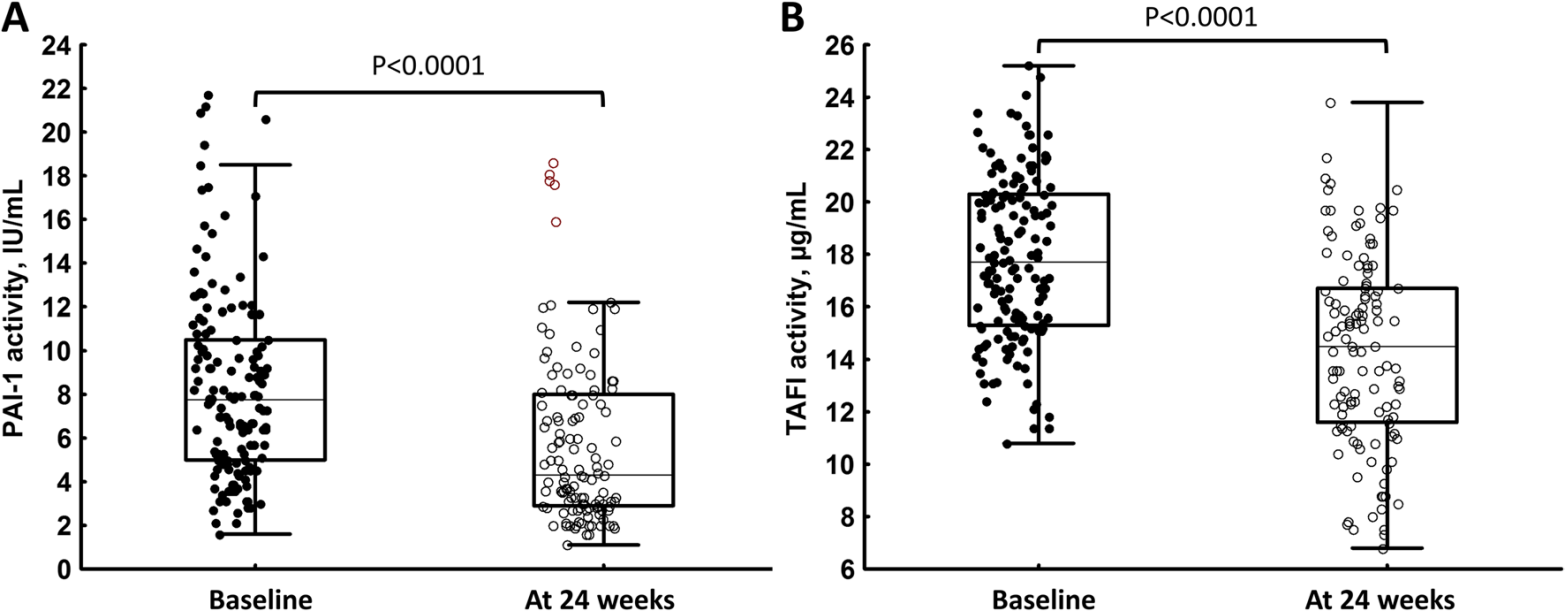
Plasminogen activator inhibitor type 1 (PAI-1) activity (panel A) and thrombin activatable fibrinolysis inhibitor (TAFI) activity (panel B) in all women on hormone therapy at baseline and at the end of the study

Importantly, in the whole group HT resulted in decreased activity of PAI-1 (-42.5%) and TAFI (-17.3%) compared to baseline values (Table 2, Fig. 3), without any changes in their antigen concentrations (Table 2). There were no similar differences in controls (Table 2). Of note, CLT remained unchanged in control women after 24 weeks compared to baseline (96 [88–106] vs 97 [86–111] min, *p* = 0.62). The ultra-low dose and standard HT groups did not differ with regard to on-treatment PAI-1 activity (4.4 [3.0–6.8] vs 4.7 [3.1–10.2] IU/ml) and TAFI activity (14.3 [11.6–17.3] vs. 15.1 [12–16.3] %), like CLT (95 [78–105] vs. 98 [91–195] min; all *p* > 0.05). We found no intergroup differences in α_2_-antiplasmin (103 [94–112] vs. 103 [89–113]%, *p* = 0.95) and plasminogen (107 [94–112] vs. 101 [96–127]%, *p* = 0.63).

**Table 2:** Hemostatic variables in relation to baseline PC concentration at 6-month follow-up

	Control		*P*-value	HT women		*P*-value	PC top quartile in the HT group		*P*-value
Variables	Baseline *N* = 50	At 6 months *N* = 41		Baseline *N* = 100	At 6 months *N* = 81		Baseline *N* = 25	At 6 months *N* = 21	
Antithrombin, %	92 [81–101]	90 [83–95]	0.28	91 [85–101]	91 [86–97]	0.75	90 [85–102]	89 [83–93]	0.60
Fibrinogen, g/L	3.5 [2.9–3.8]	3.4 [2.8–3.4]	0.68	3.5 [3.0–4.0]	3.4 [2.9–4.1]	0.91	3.6 [3.1–4.1]	3.5 [3.0–4.0]	0.87
FVIII, %	134 [113–149]	125 [104–125]	0.19	129 [114–151]	151 [126–175]	< 0.001	125 [112–156]	146 [126–170]	0.05
Plasminogen activity, %	99 [91–108]	101 [93–119]	0.36	99 [89–108]	102 [96–115]	0.062	97 [88–103]	101 [97–120]	0.85
PAI-1 activity, IU/mL	6.7 [4.9–9.7]	6.2 [4.7–7.6]	0.20	8.0 [5.0–11.5]	4.6 [3.1–8.0]	< 0.0001	13.3 [7.9–20.2]	4.1 [3.2–7.3]	0.0036
PAI-1 antigen, ng/mL	29 [23.7–33.1]	27.4 [24.9–29.5]	0.44	27.1 [21.2–30.5]	25.6 [21.8–28.5]	0.27	26.4 [20.4–28.9]	24.7 [22.2–28.0]	0.16
TAFI activity, µg/mL	17.3 [15.3–20.1]	16.9 [15–19.7]	0.76	17.9 [15.4–20.3]	14.8 [12.0–16.9]	< 0.0001	21.3 [18.2–24.2]	15.8 [13.1–18.9]	0.009
TAFI antigen, %	99 [86–113]	102 [92–112]	0.89	103 [89–118]	109 [96–122]	0.067	103 [98–122]	109 [97–132]	0.07
α2-antiplasmin, %	102 [94–113]	99 [89–107]	0.34	101 [93–113]	103 [93–112]	0.53	101 [89–112]	101 [94–111]	0.30
F1 + 2, pM	230 [203–269]	218 [188–250]	0.90	245 [201–293]	242 [202–299]	0.15	253 [223–301]	241 [193–290]	0.84
Lag phase, min	2.7 [2.3–3.1]	2.6 [2.2–3.4]	0.50	2.8 [2.3–3.2]	3.0 [2.6–3.5]	0.08	2.7 [2.3–3.2]	3.3 [2.7–3.7]	0.41
Peak thrombin, nM	285 [255–313]	277 [245–290]	0.67	279 [236–239]	261 [210–311]	0.48	266 [227–306]	244 [215–298]	0.43
ETP, nM × min	1577 [1427–1794]	1598 [1436–1783]	0.97	1531 [1374–1690]	1598 [1441–1883]	0.052	1593 [1346–1700]	1619 [1343–1883]	0.77

Abbreviations: see Table 1

On-treatment CLT tended to be shorter compared to baseline (96 [84–105] vs. 100 [85–111] min, *p* = 0.053). However, solely in HT patients with the highest PC levels on admission, we observed significantly shortened CLT during follow-up compared to baseline (103 [84–109] vs. 107 [97–117] min, *p* = 0.039), along with reduced PAI-1 (-69%) and TAFI activity (-26%) without any other differences (Table 2). Importantly, on-treatment CLT was shorter in patients with the highest PC compared to controls with the highest PC (103 [84–109] vs. 109 [90–119] min, *p* = 0.026). After 24 weeks, associations of on-treatment PC with CLT were not observed in the whole HT group (*p* = 0.73) as well as in patients using different HT regimens (for reduced dose, *p* = 0.81 and *p* = 0.07 for standard dose). Moreover, there were no associations of on-treatment PC with F1 + 2 prothrombin fragments, ETP or peak thrombin (all *p* > 0.05).

## Discussion

To the best of our knowledge, this is the first report to demonstrate that in postmenopausal women prior to HT enhanced PC is associated with reduced plasma fibrinolytic capacity, which might contribute to the increased risk of thromboembolic events observed in this group of patients. Moreover, the current study showed reduced PC levels after 24 weeks of HT use, regardless of its regimen. The current report also suggests a profibrinolytic effect of HT, associated at least in part, with the ability of HT to decrease the activity of the key fibrinolysis inhibitors, namely PAI-1 and TAFI. The present findings provide new insights into the role of enhanced protein carbonylation in regulating fibrinolysis after menopause and the profibrinolytic effects of oral HT therapy in relation to PC levels.

Aging is related to an unfavorable progressive modification in fibrin clot properties and alterations in activity or levels of coagulation factors leading to increased thrombotic tendency [28]. These effects are mediated via different mechanisms, including enhanced oxidative stress and PC is its specific and stable marker [28]. Fibrinogen is especially susceptible to carbonylation [6, 7]. Post-translational oxidative modifications affect fibrinogen function and therefore can alter clot formation, modify clot structure and susceptibility to lysis, leading to an increased risk of thrombosis [7]. Several studies reported changes in fibrin clot properties after protein carbonylation reflected by decreased stiffness [6, 29–31], permeability [6, 29, 31, 32], and increased density of fibrin clots [31, 33, 34]. However, data on PC-mediated effects on fibrinolysis are inconsistent and encompass reduced [25, 30, 31, 35] or increased [4] susceptibility to lysis, as well as no influence of PC on lysis [29, 36], which probably in part depends on the model or assay used. Other fibrinolytic proteins are known to undergo oxidative modifications, such as PAI-1 or plasminogen [38, 39], however, their role in fibrinolysis is still unclear.

We showed previously unfavorably modified fibrin clot characteristics in postmenopausal women compared to the premenopausal ones [37], however, the mechanism of this phenomenon has not been characterized. The current study suggests that after menopause decreased plasma clot susceptibility to lysis may at least in part result from increased carbonylation of fibrinogen and fibrinolytic proteins, such as PAI-1, TAFI, and plasminogen, which activity tended to be lower in patients with high PC levels. It has been shown that the carbonylation of PAI-1 can affect its activity and stability [38], which may affect the regulation of hemostasis, fibrinolysis, and other processes related to blood clotting. However, the specific consequences of PAI-1 carbonylation may depend on environmental conditions and have not been established [38]. It was also suggested that carbonylation of TAFI may exert similar effects on protein activity and/or stability [39]. Nevertheless, no changes in PAI-1 and TAFI were observed in controls after follow-up. Further studies are needed to elucidate mechanisms linking enhanced PC and hypofibrinolysis observed in menopausal women and the potential of HT to modulate such associations.

Although HT is known to increase FVIII, F1 + 2, and ETP [22], in women with the highest PC we observed only a tendency to increased thrombin generation capacity and similar F1 + 2, although its levels were above the upper limit of the reference range. It may suggest no significant influence of carbonylation on thrombin generation per se in this group of patients. It might be speculated that carbonylation of plasma proteins accompanied by increased activity of fibrinolysis inhibitors contribute to hypofibrinolysis. Since our study did not assess fibrinogen carbonylation further studies are required to elucidate the extent of this process in postmenopausal women.

The separate issue that deserves a comment is estrogens’ involvement in oxidative stress. There is a controversy whether estrogens have pro- or antioxidant effects [20, 21, 40–43]. The apparent discrepancy is related to the chemical heterogeneity in the estrogen family, as well as, their concentration and the environment in which they are found [40]. Growing evidence has shown that estrogens reduce the ROS production and consequently affect oxidative stress both in vitro and in vivo [41, 42]. On the other hand, this antioxidant hypothesis stays in contradiction with the proposed pro-oxidant mechanism revealed in breast cancer development [43]. Nevertheless, our report confirms the concept that HT may contribute to reduced PC, which is in line with the two studies that showed decreased PC levels after both transdermal and oral routes of HT when compared with the controls [20]. Our study expands the current knowledge on HT effects by demonstrating that it can, at least in part, increase efficiency of fibrinolysis. Even if CLT is determined by PC only in 15%, PC reduction on HT may contribute to shorter lysis time of fibrin clots. It might be speculated that in postmenopausal women changes in oxidative stress expressed as PC content can affect balance between prothrombotic and antithrombotic mechanisms, especially given the fact that in most of HT-treated postmenopausal women no thromboembolic events are observed. Further studies are needed to elucidate the impact of different types of progestogens on PC. Moreover, we previously observed a more prothrombotic fibrin clot phenotype in postmenopausal women as compared to the healthy controls [22, 44], which together with the current findings, suggests that decreased protein carbonylation can favorably modify not only fibrinolysis but also clot structure. Several studies along with the Cochrane analysis [45] showed an increased risk of thrombosis related to HT after at least one year of treatment. In our study with no thromboembolic events after 6 months of HT, we have shown shortened CLT along with a tendency to higher thrombin generation and increased FVIII levels. On the other hand, as stated above estrogens exert antioxidant effects and are able to decrease protein carbonylation. Precise mechanisms underlying the regulation of fibrin formation and degradation in postmenopausal women on HT remain to be established.

Our study has several limitations. First, the size of the study was limited, especially in the subgroup analysis. However, our research used a random allocation of women to the three groups. Moreover, the study was hypothesis-generating and the presented associations do not necessarily mean the cause-effect relationship, therefore our results should be interpreted with caution. Second, our study excluded women with comorbidities known to increase PC and negatively alter fibrinolysis, in particular diabetes [29], which may limit the applicability of our findings to the general population of postmenopausal women. Third, we did not assess all enzymes involved in fibrinolysis, such as tPA or urokinase-type PA. Moreover, we did not perform in vitro experiments to show which proteins were affected by carbonylation in postmenopausal women, in particular PAI-1 and TAFI. Fourth, in treated women, therapeutic compliance was assessed only through self-reporting, therefore we cannot exclude that HT was not taken regularly by all subjects.

## Conclusion

Our randomized study suggests that in postmenopausal women enhanced PC is associated with hypofibrinolysis accompanied by increased activity of PAI-1 and TAFI. After 24 weeks of HT PC levels are lowered along with enhanced fibrinolysis. Mechanistic studies involving all enzymes regulating fibrinolysis, including an assessment of their carbonylation, are needed to support our observation.

## Data Availability

Data are available on request.
